# Development and validation of a protocol for cell line identification by MALDI-TOF MS

**DOI:** 10.1186/1753-6561-5-S8-P45

**Published:** 2011-11-22

**Authors:** Guido Vogel, André Strauss, Bernard Jenni, Dominik Ziegler, Eric Dumermuth, Sylvie Antz, Claudia Bardouille, Beat Wipf, Christian Miscenic, Georg Schmid, Valentin Pflüger

**Affiliations:** 1 Mabritec AG, 4125 Riehen, Switzerland; 2 Novartis Pharma AG , 4056 Basel, Switzerland; 3 F. Hoffmann-La Roche AG, 4070 Basel, Switzerland

## Summary

Misidentification or cross-contamination of cell lines used in biotechnology or diagnostic settings are a challenge for laboratories and cell culture repositories. Masters *et al*. [1] among others reported the occurrence of large numbers of unrecognized and unreported misidentification or cross-contamination of cell lines. Current methods for the authentication of cell lines such as karyotyping, 2D-gel-electrophoresis, restriction fragment length polymorphism (RFLP) or short tandem repeats (STR) are expensive, labor intensive and not routinely applied. In the last decade MALDI-TOF MS became a powerful tool for the rapid and cost effective identification and taxonomic classification of microorganisms directly from whole cell extracts. We adapted this protein fingerprinting approach for a fast species identification of eukaryotic cell lines and established as well as validated a reference database.

In addition we demonstrate that this new approach has a potential for the rapid characterization of recombinant protein expression systems. Accurate protein expression and the determination of the molecular mass of the recombinant expressed proteins (20-120kDa) can directly be analyzed from stable transfected and virus infected cultures.

## Materials and methods

1 ml of fresh culture was transferred into an Eppendorf tube and washed once with 1x PBS. The pellet was resuspended in 70% EtOH for transport and storage at RT. 1µl of the cell pellet was transferred with a plastic loop to a new Eppendorf tube and mixed with 20µl formic acid (10%). The suspension was mixed with 40µl of saturated sinapinic acid matrix. 1µl of sample suspension was spotted on a steel target plate in duplicates. Spectra were acquired on Shimadzu Confidence MALDI-TOF MS in linear mode in a mass range from 2-50kDa. Peak lists were imported into SARAMIS software for Marker pattern definition and automated identification.

## Results

**Figure 1:**
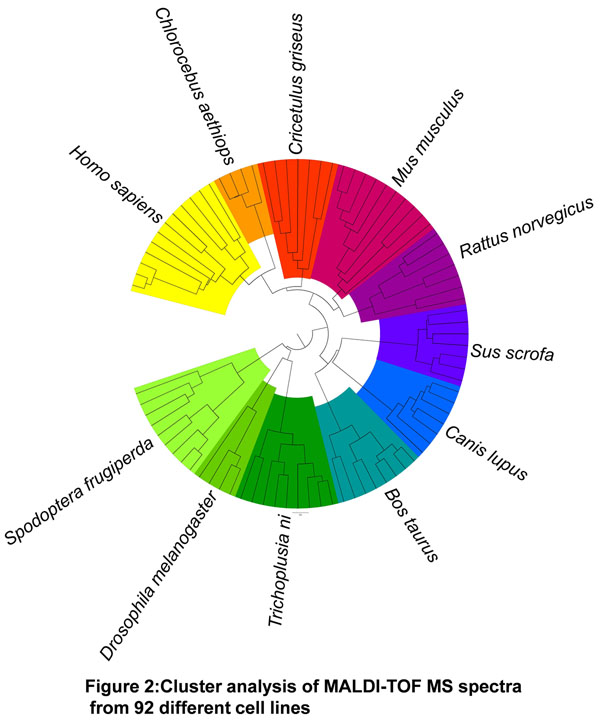
Cluster analysis of MALDI-TOF MS spectra from 92 different cell lines

In a first phase we analyzed 146 different eukaryotic cell lines derived from 11 different taxa (Figure 1), including common lines from culture collections as well as customer specific in-house lines. Marker patterns were established for these 11 taxa and validated in a blinded study with 9 mammalian and 48 insect cell lines. All 57 test samples were correctly identified on the species level. Even two intentionally mixed samples were assigned to the right species.

In a second phase we analyzed more than 178 mammalian and 240 insect cell lines for identity authentication from different customers in order to evaluate the usefulness of this service. Out of 418 samples analyzed, we revealed 6 samples with either wrong identities, strongly altered or unknown mass profiles (Table 1).

**Table 1:** Example of problematic samples

sample name	expected species	MALDI-TOF MS results
**Sf9**	*Spodoptera frugiperda*	*Trichoplusia ni*
**HepG2**	*Homo sapiens*	*Mus musculus*
**SK-MEL-30**	*Homo sapiens*	*Homo sapiens* profile with strongly altered mass composition: contamination possible
**CHO-K1**	*Cricetulus griseus*	unknown mass profile
**J558L**	*Mus musculus*	*Mus musculus* profile with strongly altered mass composition: contamination possible
**WS1**	*Homo sapiens*	unknown mass profile

When necessary cell lines were also analyzed for their expression profile after infection or transfection with viral vectors. It was possible to determine precise masses of overexpressed proteins as compared to controlled samples. For these additional experiments, no further sample preparation was necessary.

## Conclusion

We developed a fast, simple and accurate method with high throughput capacity for the authentication of cell lines. Since the construction of customer specific mass profile libraries is straightforward, we propose this approach as an additional method for routine cell line authentication in biotechnology settings.

Further we demonstrated that MALDI-TOF MS is also a fast and simple tool for the characterization of eukaryotic protein expression systems, especially for the determination of a precise mass.
